# Exploring the Modulatory Effect of High-Fat Nutrition on Lipopolysaccharide-Induced Acute Lung Injury in Vagotomized Rats and the Role of the Vagus Nerve

**DOI:** 10.3390/nu15102327

**Published:** 2023-05-16

**Authors:** Maarten F. J. Seesing, Henricus J. B. Janssen, Tessa C. M. Geraedts, Teus J. Weijs, Ingrid van Ark, Thea Leusink-Muis, Gert Folkerts, Johan Garssen, Jelle P. Ruurda, Grard A. P. Nieuwenhuijzen, Richard van Hillegersberg, Misha D. P. Luyer

**Affiliations:** 1 Department of Surgery, University Medical Center Utrecht, Utrecht University, 3584 Utrecht, The Netherlands; 2 Department of Surgery, Catharina Hospital, 5623 Eindhoven, The Netherlands; thijs.janssen@catharinaziekenhuis.nl (H.J.B.J.);; 3 Division of Pharmacology, Department of Pharmaceutical Sciences, Faculty of Science, Utrecht University, 3584 Utrecht, The Netherlands; 4 Danone Nutricia Research & Innovation, Immunology, 3584 Utrecht, The Netherlands

**Keywords:** vagus nerve, nerve stimulation, acetylcholine, cholecystokinin, agonist, high-fat, vagotomy, inflammation, rats

## Abstract

During esophagectomy, the vagus nerve is transected, which may add to the development of postoperative complications. The vagus nerve has been shown to attenuate inflammation and can be activated by a high-fat nutrition via the release of acetylcholine. This binds to α7 nicotinic acetylcholine receptors (α7nAChR) and inhibits α7nAChR-expressing inflammatory cells. This study investigates the role of the vagus nerve and the effect of high-fat nutrition on lipopolysaccharide (LPS)-induced lung injury in rats. Firstly, 48 rats were randomized in 4 groups as follows: sham (sparing vagus nerve), abdominal (selective) vagotomy, cervical vagotomy and cervical vagotomy with an α7nAChR-agonist. Secondly, 24 rats were randomized in 3 groups as follows: sham, sham with an α7nAChR-antagonist and cervical vagotomy with an α7nAChR-antagonist. Finally, 24 rats were randomized in 3 groups as follows: fasting, high-fat nutrition before sham and high-fat nutrition before selective vagotomy. Abdominal (selective) vagotomy did not impact histopathological lung injury (LIS) compared with the control (sham) group (*p* > 0.999). There was a trend in aggravation of LIS after cervical vagotomy (*p* = 0.051), even after an α7nAChR-agonist (*p* = 0.090). Cervical vagotomy with an α7nAChR-antagonist aggravated lung injury (*p* = 0.004). Furthermore, cervical vagotomy increased macrophages in bronchoalveolar lavage (BAL) fluid and negatively impacted pulmonary function. Other inflammatory cells, TNF-α and IL-6, in the BALF and serum were unaffected. High-fat nutrition reduced LIS after sham (*p* = 0.012) and selective vagotomy (*p* = 0.002) compared to fasting. vagotomy. This study underlines the role of the vagus nerve in lung injury and shows that vagus nerve stimulation using high-fat nutrition is effective in reducing lung injury, even after selective vagotomy.

## 1. Introduction

Stimulation of the vagus nerve reduces inflammatory responses upon several stimuli [1,2,3,4,5,6]. Upon stimulation, efferent vagus nerve endings release acetylcholine (ACh). Acetylcholine can bind to the α7 nicotinic acetylcholine receptor (α7nAChR) found on inflammatory cells, thereby inhibiting the release of pro-inflammatory cytokines (e.g., TNF-α and IL-6) which attenuates systemic and local inflammatory responses [7,8,9,10]. It can be stimulated electrically, via pharmacological agents and via high-fat enteral nutrition [1,2,3,4,5,6]. Lipids activate the cholinergic anti-inflammatory pathway (CAIP) via cholecystokinin (CCK)-receptors located centrally or on peripheral vagal afferents [3]. The activation of CCK-receptors triggers vagal efferents to release ACh and subsequently inhibit α7nAChR-expressing inflammatory cells [3].

The vagus nerve is also important for bronchial smooth muscle contraction, vascular congestion, mucus secretion and mucosal swelling via vagus efferents [7,8,11,12,13,14]. Furthermore, the vagus nerve receives feedback from the lung through its afferent fibers that, amongst others, participate in mucosal mast cell activation and the cough reflex to protect against toxins, microorganisms and other foreign materials [15,16,17], as well as in regulating pulmonary stretch receptors to prevent hyperinflation through the Hering–Breuer reflex [12,18,19].

During an esophagectomy, the vagus nerve needs to be transected as part of the oncological procedure [11,13,14,20,21]. Since the vagus nerve is both involved in the CAIP and in regulating the aforementioned physiological functions in the lung through its pulmonary branches, the vagotomy may be an important factor in the development of pulmonary complications following esophagectomy. Although several factors have been associated with postoperative (pulmonary) complications [22,23,24,25,26], the exact pathogenesis is not fully understood [27,28,29]. As pulmonary complications often lead to a prolonged length of hospital and ICU stay, increased hospital and ICU readmission rate and even mortality, the role of the vagotomy in pulmonary complications and lung injury following esophagectomy could be important [13,30,31,32].

Hence, this study aimed to investigate the role of the α7nAChR and the effect of a vagotomy on lipopolysaccharide (LPS)-induced acute lung injury and pulmonary function. Additionally, the effect of high-fat nutrition after sham and selective vagotomy was investigated.

## 2. Materials and Methods

### 2.1. Animals

Male Sprague Dawley rats, weighing 300 to 350 g, purchased from Charles River Laboratories (Erkrath, Germany) were used. The rats were housed in a temperature- and humidity-controlled room on a 12 h light/dark cycle. In phase one of the experiments, food was provided according to a standardized regimen. In phase two, food and water were provided ad libitum. All animal experiments were conducted in compliance with the Guidelines of the Ethical Committee on the Use of Laboratory Animals of the Utrecht University and was approved by the Central Authority for Scientific Procedures on Animals (CCD).

### 2.2. Surgical Procedure

**Figure 1:**
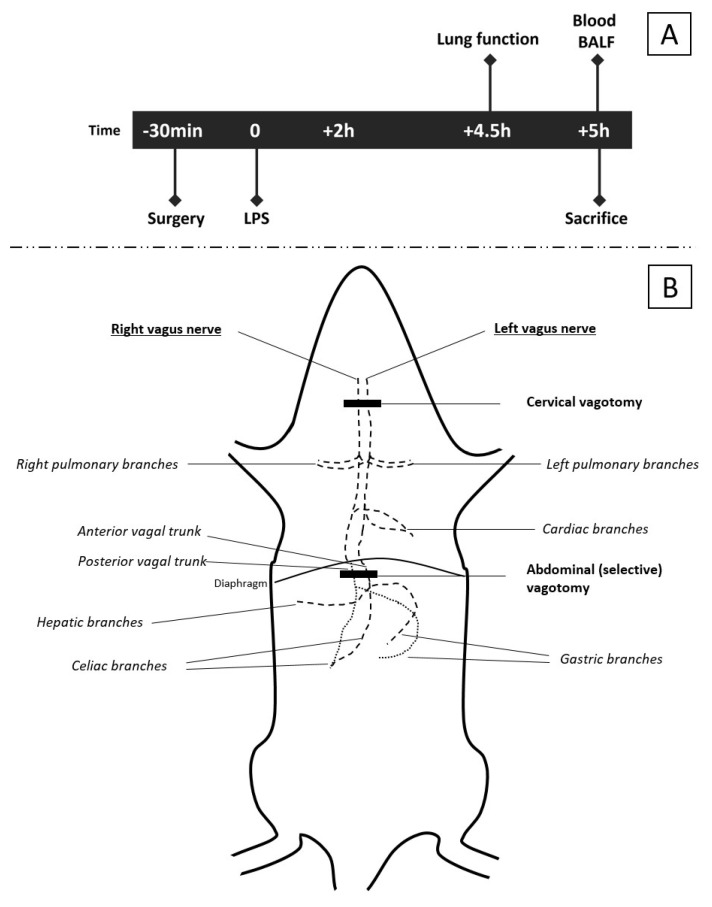
**Schematic overview of experiments.** The timeline (**A**) of interventions and measurements is applicable to all three models. The specific surgical procedures and intervention are described in detail in the methods section. A gross schematic representation of the vagus nerve (**B**) and level of the vagotomy [7,8,34,35]. The cervical vagotomy was performed at the level of the trachea after division of the submaxillary glands. The abdominal (selective) vagotomy was performed by transecting the anterior and posterior trunks subdiaphragmatically. In sham rats (controls), the vagus nerves were also exposed, but spared.

Rats were anesthetized intraperitoneally with urethane (10%, 2 g/kg, U2500, Sigma-Aldrich, Zwijndrecht, The Netherlands). Anesthetic depth was assessed every 30 min by the absence of protective eye (corneal) reflexes and withdrawal reflexes after toe pinch. Rectal temperature was monitored throughout the experiment and was kept between 36.5 °C and 37.5 °C using a heating pad and blankets. During the surgical procedure, rats were tracheotomized and a tracheal cannula was placed and fixed with ligatures. Subsequently, rats were administered 100 μL—containing 50 μg/kg lipopolysaccharide (LPS) (Sigma-Aldrich, Zwijndrecht, The Netherlands)—intratracheally through the previously placed tracheal cannula, to induce acute lung injury. Three experimental procedures (Model A, B and C) were designed to investigate the role of the vagus nerve and high-fat nutrition on LPS-induced lung injury. A schematic overview of the experiments is shown in Figure 1A and applies to all models. Rats were euthanized 300 min after LPS administration by an overdose of intraperitoneally injected pentobarbital (150 mg/kg) (Euthesate™, Ceva Santé Animale, Naaldwijk, The Netherlands). Pulmonary tissue, bronchoalveolar fluid (BALF) and blood samples were isolated and examined in accordance with the American Thoracic Society guidelines [33].

### 2.3. Experimental Models

#### 2.3.1. Model A: Effect of Selectively Sparing Vagus Nerve on Lung Injury and Function

Model A was designed to investigate the effect of the level of the vagotomy on inflammation and lung injury and investigate the role of α7nACh receptors herein. In this experiment, 48 rats were randomized into 4 groups (N = 12 each). A sham (control) group underwent a cervical and abdominal sham procedure as follows: A small cervical midline incision was made, and after division of the submaxillary glands and muscles, the vagus nerve was identified bilaterally (Figure 1B). Subsequently, rats were tracheotomized and the cervical midline incision was closed. Next, a midline laparotomy incision was made, and the anterior and posterior subdiaphragmatic branches of the vagus nerve were exposed by mobilizing the left liver lobe, after which the abdomen was closed (Figure 1B). The abdominal vagotomy group underwent the same procedure, but after identification of the anterior and posterior branches, both were transected subdiaphragmatically, while sparing the cervical vagus (Figure 1B). This modelled for a selective (abdominal) vagotomy (i.e., sparing the pulmonary vagal branches). A cervical vagotomy group underwent the same procedure as the control and selective (abdominal) vagotomy groups; however, after identification of the cervical vagus nerve, it was transected bilaterally, which modelled for transecting the pulmonary vagal branches (cervical vagotomy group), Figure 1B. Lastly, one group underwent the same procedure as the cervical vagotomy group; however, after closing the abdominal incision, an α7nAChR-agonist (GTS-21; 4 mg/kg, Sigma-Aldrich, Zwijndrecht, The Netherlands) was injected intraperitoneally (agonist vagotomy group).

#### 2.3.2. Model B: Further Exploring the Role of the α7nAChR

Model B was designed to further explore the role of the vagus nerve and test whether lung injury can be aggravated by administration of an α7nAChR-antagonist to inhibit the CAIP or pulmonary functions, or if other pathways could be involved. In this experiment, 24 rats were randomized into 3 groups (N = 8 each). A sham (control) group underwent a cervical sham procedure similar to the sham group of Model A (Figure 1B); however, no abdominal incision was made. The second group received an α7nAChR-antagonist (Chlorisondamine diiodide, 0.125 mg/kg, Sigma-Aldrich, Zwijndrecht, The Netherlands) intravenously via the right internal jugular vein prior to performing the same cervical sham procedure (antagonist sham group). The third group underwent the same procedure, but after administration of the α7nAChR-antagonist, the cervical vagus nerve was transected bilaterally (antagonist vagotomy group).

#### 2.3.3. Model C: Vagus Nerve Stimulation through High-Fat Enteral Nutrition

Model C was designed to investigate the effect of high-fat nutrition after selective vagotomy. This model was performed since a feeding regimen could be easier to implement in a clinical setting than α7nAChR-agonists as a stimulating agent. Rats were either starved overnight (i.e., fasted for 18 h), or received high-fat nutrition according to a standardized regimen before the surgical procedure. High-fat nutrition was administered at three time points as follows: at T = −18 h, an oral gavage of 3 mL high-fat nutrition; at T = −2 h, 1 mL of high-fat nutrition; and at T = −45 min, another oral gavage of 1 mL of high-fat nutrition. At T = 0 (i.e., directly after surgery), all rats received LPS similar to the previous models. High-fat nutrition contained 50.4 energy percent (en%) fat—of which 30 en% were phospholipids—8.7 en% protein, and 40.9 en% carbohydrates (Sigma-Aldrich, Zwijndrecht, The Netherlands). The lipid source was soy lecithin. Omega-3 and omega-6 fatty acids constituted less than 5 wt% (<5 g/100 mL). Proteins were derived from lean milk powder containing 80% casein and 20% whey protein. The source for carbohydrates was a mixture of sucrose and maltodextrins (Glucidex 19DE). In this experiment, 24 rats were randomized into 3 groups (N = 8 each). The sham (control) group was fasted for 18 h and subsequently underwent an abdominal sham procedure as previously described. The other two groups received high-fat enteral nutrition according to the standardized regimen described above. One of these groups (HF-sham) underwent the same abdominal sham procedure as the control group. The other group underwent a selective (abdominal) vagotomy (HF-selective vagotomy).

### 2.4. Pulmonary Function Measurement

Rats were placed in a temperature-controlled plethysmograph (body temperature was kept at 37 °C) in which they were ventilated (ventilation rate: 90 beats/min; volume 2 mL/beat) at 270 min after LPS administration. The previously placed tracheostomy was replaced by a small catheter that was connected to a pressure transducer fixed on the plethysmograph box (EMKA Technologies, Paris, France). Transpulmonary pressure (cm H_2_O) was determined by measuring pressure differences in the cannula in the trachea. Increasing doses of methacholine (acetyl-β-methyl-choline chloride, Sigma-Aldrich, Zwijndrecht, The Netherlands) (0.37–50 mg/mL, 10% puff for 10 s) were administered by aerosol generated in a nebulizer (EMKA Technologies, Paris, France) connected in between the plethysmograph and the ventilator (EMKA Technologies, Paris, France). After the first dose of methacholine, pulmonary resistance (in cm H_2_O/(mL·s^−1^)) was measured for 3 min. This procedure was repeated for all doses. Subsequently, rats were sacrificed (at 300 min as previously described).

### 2.5. Bronchoalveolar Lavage (BAL)

After the rats were sacrificed, the thoracic cavity was opened, and both lungs were taken out. The right main bronchus was canulated, and the right lungs were lavaged with 2 mL of pyrogen-free saline (0.9% NaCl, 37 °C) supplemented with protease inhibitor cocktail tablet (Complete Mini, Roche Diagnostics, Mannheim, Germany). The supernatant of the first mL was used for cytokine measurement. Afterwards, the right lungs were lavaged 2 times with 2 mL saline solution (0.9% NaCl, 37 °C). The BAL fluid (BALF) was centrifuged (300× *g*, at 4 °C, 5 min) and pellets of the 3 lavages were pooled. Total number of BALF cells were counted using a Bürker-Türk bright-line counting chamber (magnification 100×) (Karl Hecht Assistant KG, Sondheim/Rohm, Germany). For differential cell counts, cytospin preparations were made and stained with Diff-Quick (Merz and Dade A.G., Düdingen, Switzerland). After coding, all cytospin preparations were evaluated by 2 independent observers using oil immersion microscopy (Leitz Optilux, Leica, Wetzlar, Germany). Number of macrophages, lymphocytes and neutrophils were scored based on standard morphology. At least 200 cells per cytospin preparation were counted, and the absolute number of each cell type was calculated.

### 2.6. Preparation of Lung Homogenates

After the right lungs were lavaged, the samples (150 mg/mL) were lysed on ice (using a lysis buffer: 200 mM NaCl, 5 mM EDTA, 10 mM Tris, 10% glycerine, 1 mM PMSF, 1 μg/mL leupeptin and 28 μg/mL aprotinin (Sigma-Aldrich)) and homogenized. Lung samples were then centrifuged twice (1500× *g* at 4 °C for 15 min), and supernatants were collected and stored at −20 °C until further analysis of TNF-α and IL-6 levels.

### 2.7. Lung Histology

The left lungs were fixed with 10% formalin infusion for 24 h and embedded in paraffin after fixation. Subsequently, 5 μm thick lung sections were cut (Leica, model RM2165, Germany) and stained with haematoxylin/eosin (H&E). Photomicrographs were taken with an Eclipse E800M microscope (Nikon Instruments Inc., Amstelveen, The Netherlands) equipped with a Nikon DXM 1200 digital camera (Nikon Instruments Inc. The Netherlands). Histopathological lung injury was determined in the H&E section according to the guidelines of the American Thoracic Society, in which scores range from 0 to 1, with higher scores indicating more severe lung injury.

### 2.8. ELISA

Blood samples were obtained via cardiac puncture after rats were sacrificed. Twenty µL of blood was used to count the total numbers of leukocytes and a blood smear was made to determine the cell count of various white blood cells. The rest of the blood was centrifuged (14,000× *g*, Room Temperature, 5 min). Plasma was collected and samples were kept at −20 °C. Subsequently, IL-6 (BMS625) and TNF-α (BMS622) were measured in plasma and lung homogenates with a Ready-SET-Go!^®^ ELISA kit (eBioscience, San Diego, CA, USA), and concentrations were expressed as pg/mL.

### 2.9. Statistics

Statistical analyses were performed using IBM SPSS Statistics for Windows, Version 29.0 (Armonk, NY, USA: IBM Corp). Data were tested for normal distribution using the Shapiro–Wilk test. Depending on data normality, numerical data are presented as means with standard deviation (±SD) or medians with interquartile range (IQR; lower quartile–upper quartile). Similarly, a one-way analysis of variance (ANOVA) or a non-parametric test (i.e., Kruskal–Wallis test) was used for statistical comparisons between data points for multiple groups. Differences between two groups were analyzed using the *t*-test (unpaired) or Mann–Whitney U test. A *p*-value of < 0.05 was considered statistically significant. The *p*-values were corrected for multiple comparisons using the Bonferroni method.

## 3. Results

### 3.1. Model A: Selectively Sparing Vagus Nerve

**Figure 2:**
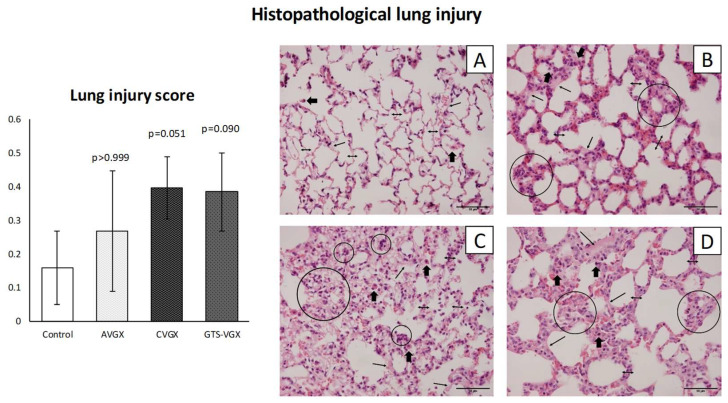
**Model A. Lung injury score.** There was an increased trend in histopathological lung injury after a cervical vagotomy (even after an α7nAChR-gonist), but not selective vagotomy. Histological sections of the lungs were stained with H&E at ×400 magnification (scale bars represent 50 µm). Control (**A**): alveolar walls are relatively thin (double headed arrows), and the alveoli contain occasional alveolar macrophages (thick arrows); (**B**) AVGX: somewhat thickened alveolar walls infiltrated with macrophages and neutrophils (circles); (**C**) CVGX and (**D**) GTS-VGX: alveolar walls are thickened with intramural macrophages and neutrophils. Some hyaline membranes and proteinaceous debris filling the airspaces can be observed (small single-headed arrow). Values are medians with interquartile range. *p*-values as compared to the control group (after Bonferroni correction). Abbreviations: H&E, *hematoxylin and eosin*; LIS, *lung injury score*; Control, *sham procedure*; AVGX, *abdominal (selective) vagotomy*; CVGX, *cervical vagotomy*; GTS-VGX, *cervical vagotomy with an α7nAChR-agonist (GTS-21)*. N = 4 in each group.

A selective vagotomy did not impact the histopathological lung injury score (LIS) when compared with the control (sham) group (median 0.260 (IQR 0.101–0.442) vs. 0.115 (IQR 0.089–0.273) in controls, *p* > 0.999), as shown in Figure 2. Contrarily, after a cervical vagotomy, the LIS was relatively higher (median 0.368 (IQR 0.329–0.493), *p* = 0.051), but this did not reach statistical significance. In the cervical vagotomy group that received an α7nAChR-agonist, the median LIS was 0.349 (IQR 0.299–0.505) (*p* = 0.090).

**Figure 3:**
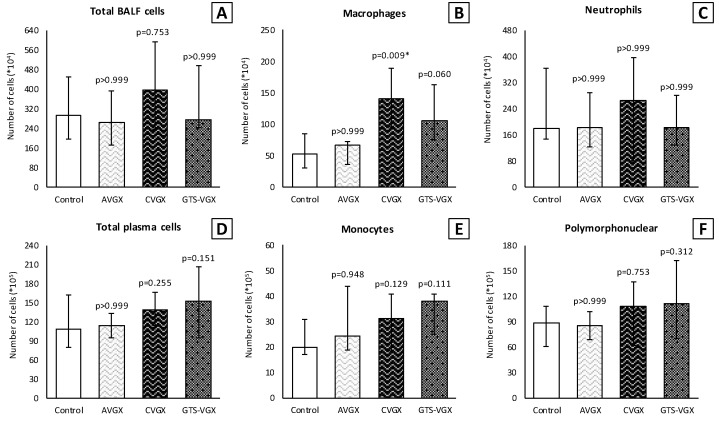
**Model A. Number of inflammatory cells in BALF (×10^4^) and plasma (×10^5^).** Cell count of (**B**) macrophages was higher in the CVGX group and there was an increased trend in the GTS-VGX group. There were no statistically significant differences between the groups in (**A**) total cell count or (**C**) neutrophils in BALF. Similarly, there were no differences between the groups in (**D**) total cell count, (**E**) monocytes and (**F**) polymorphonuclear cells in plasma. Values are medians with interquartile range. *p*-values as compared to the control group (after Bonferroni correction); a *p*-value < 0.05 was considered statistically significant (*). Abbreviations: BALF, *broncho-alveolar lavage fluid*; Control, (N = 11) *sham procedure*; AVGX, (N = 12) *abdominal vagotomy*; CVGX, (N = 10) *cervical vagotomy*; GTS-VGX, (N = 12) *cervical vagotomy with an α7nAChR-agonist (GTS-21)*.

**Figure 4:**
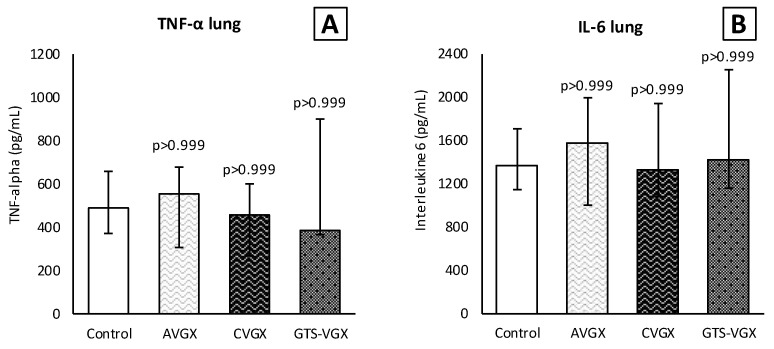
**Model A. Cytokines in lung homogenates.** Levels (pg/mL) of (**A**) TNF-α and (**B**) IL-6 in lung homogenates did not differ between the groups. Values are medians with interquartile range. *p*-values as compared to the control group (after Bonferroni correction). Abbreviations: TNF, *tumor necrosis factor*; IL, *interleukin*; Control, (N = 12) *sham procedure*; AVGX, (N = 11) *abdominal (selective) vagotomy*; CVGX, (N = 12) *cervical vagotomy*; GTS-VGX, (N = 11) *cervical vagotomy with an α7nAChR-agonist (GTS-21)*.

Inflammatory cell count (×10^4^) in the BALF was similar between the control and selective vagotomy group (Figure 3). Respectively, the median total cell count was 302 (IQR 201–460) vs. 263 (IQR 171–393) (*p* > 0.999). The median number of macrophages was 57 (IQR 30–94) vs. 67 (IQR 36–72) (*p* > 0.999), and neutrophils 207 (IQR 144–386) vs. 183 (IQR 122–290) (*p* > 0.999). Contrarily, the number of macrophages (×10^4^) increased after a cervical vagotomy (median 141 (IQR 96–189); *p* = 0.009) and a cervical vagotomy with an α7nAChR-agonist (median 106 (IQR 75–163); *p* = 0.060) compared to the control group. The total cell count and neutrophils in the BALF did not differ between the groups (Figure 3). In the plasma, the inflammatory cells did not differ between the control and vagotomy groups (Figure 3). The levels of TNF-α and IL-6 in the lung homogenates were also similar (Figure 4).

**Figure 5:**
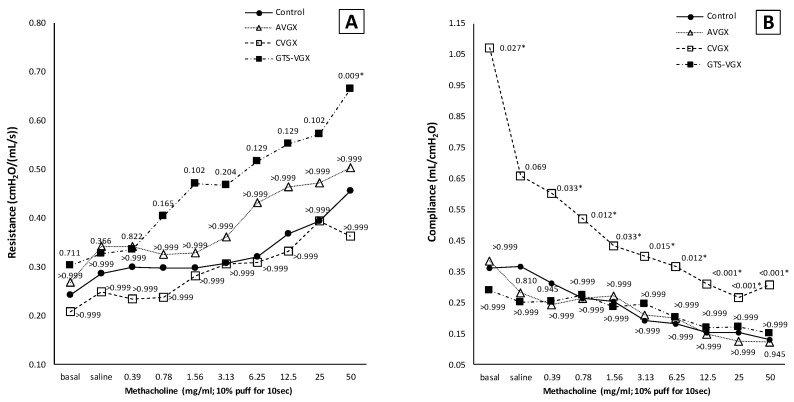
**Model A. Pulmonary function.** A vagotomy—irrespective of the level at which it was transected—did not affect (**A**) airway resistance. However, there was a trend toward an increased airway resistance after GTS-21, while lung compliance (**B**) was unaffected in this group. Contrarily, in the CVGX group, dynamic lung compliance was increased compared to a sham procedure and selective vagotomy, while airway resistance was unaffected. Values are medians (interquartile range is not shown for clarity). *p*-values as compared to the control group (after Bonferroni correction); a *p*-value < 0.05 was considered statistically significant (*). Control, (N = 10) *sham procedure*; AVGX, (N = 7) *abdominal (selective) vagotomy*; CVGX, (N = 10) *cervical vagotomy*; GTS-VGX, (N = 8) *cervical vagotomy with an α7nAChR-agonist (GTS-21)*.

Notably, a selective vagotomy did not affect pulmonary function (i.e., airway resistance and dynamic compliance) compared to the control group, whereas a cervical vagotomy—with and without an α7nAChR-agonist—inversely affected pulmonary function (Figure 5). Specifically, dynamic lung compliance (mL/cm H_2_O) was increased in the cervical vagotomy group at baseline (median 1.071 (IQR 0.383–2.037) versus 0.361 (IQR 0.313–0.479) (*p* = 0.015) in the control group) and final (methacholine 50 mg/mL) measurement (median 0.307 (IQR 0.189–0.512) versus 0.130 (IQR 0.101–0.161), *p* = 0.003), Figure 5B. Airway resistance did not differ between both groups (Figure 5A). Contrarily, airway resistance was higher after cervical vagotomy with an α7nAChR-agonist at methacholine 50 mg/mL (median 0.665 (IQR 0.585–0.808) versus 0.456 (IQR 0.332–0.536) (*p* = 0.009) in the control group), but not at baseline (Figure 5A). Dynamic lung compliance did not significantly differ between both groups (Figure 5B).

Altogether, from model A, it was concluded that a selective vagotomy did not aggravate lung injury or affect pulmonary function, whereas a cervical vagotomy aggravated lung injury and negatively impacted pulmonary function. An α7nAChR-agonist negatively impacted pulmonary function but did not affect lung injury.

### 3.2. Model B: Role of the α7nACh Receptor

**Figure 6:**
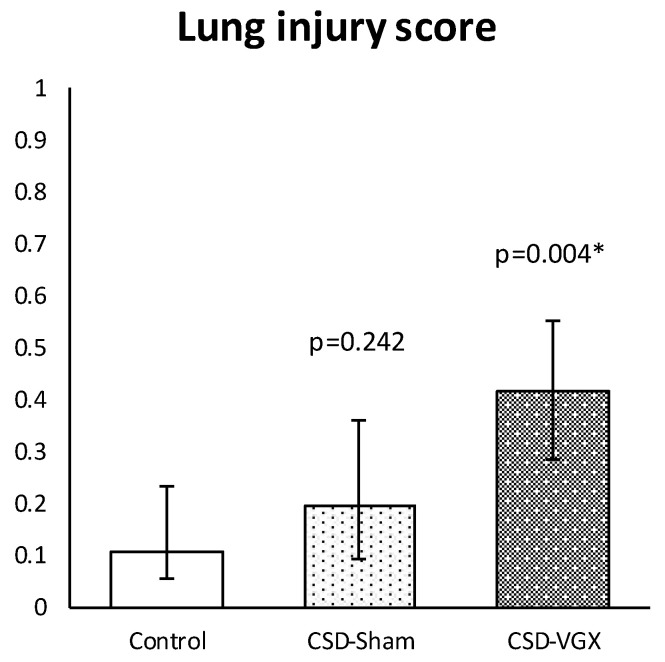
**Model B. Lung injury score.** Histopathological lung injury was similar between CSD-sham and the control group. In the CSD-vagotomy group, histopathological lung injury was more severe compared with controls and there was an increased trend compared with CSD-sham (*p* = 0.058). Values are median with interquartile range. *p*-values as compared to the control group (after Bonferroni correction); a *p*-value < 0.05 was considered statistically significant (*). Abbreviations: Control, *sham procedure*; CSD-sham, *sham cervical procedure with Chlorisondamine (α7nAChR-antagonist)*; CSD-VGX, *cervical vagotomy with Chlorisondamine*. N = 8 in each group.

**Figure 7:**
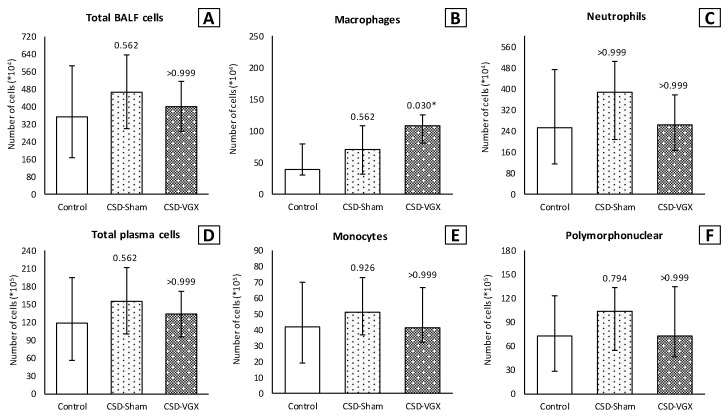
**Model B. Number of inflammatory cells in BALF (×10^4^) and plasma (×10^5^).** Cell count of (**B**) macrophages was higher in the CVGX-CSD group. There were no statistically significant differences between the groups in (**A**) total cell count and (**C**) neutrophils in BALF. Similarly, there were no differences between the groups in (**D**) total cell count, (**E**) monocytes and (**F**) polymorphonuclear cells in plasma. Values are medians with interquartile range. *p*-values as compared to the control group (after Bonferroni correction); a *p*-value < 0.05 was considered statistically significant (*). Abbreviations: BALF, *broncho-alveolar lavage fluid*; Control, (N = 8) *sham procedure*; CSD-Sham, (N = 7) *sham procedure with an α7nAChR-antagonist (Chlorisondamine)*; CSD-VGX, (N = 8) *cervical vagotomy with Chlorisondamine*.

**Figure 8:**
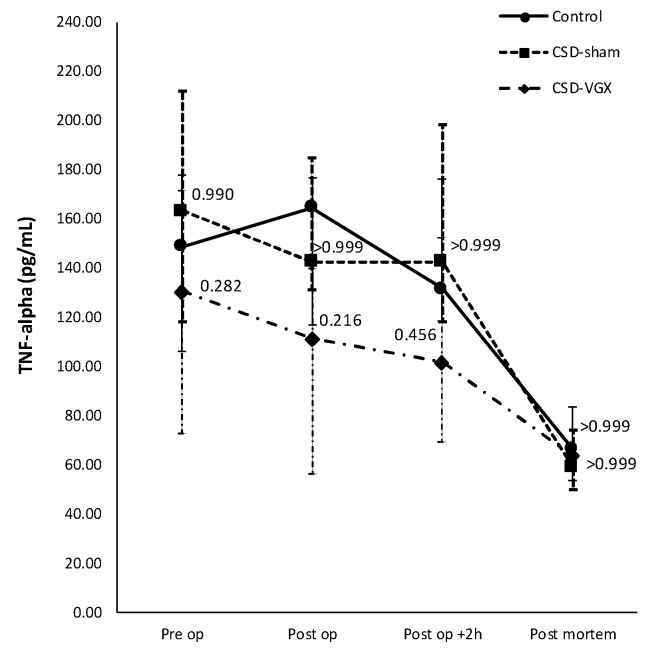
**Model B. Concentration of TNF-α over time in serum.** Levels (pg/mL) of TNF-α in serum did not differ between the groups preoperatively, directly postoperatively, at 2 h postoperatively or postmortem. Values are medians with interquartile range. *p*-values as compared to the control group (after Bonferroni correction); a *p*-value < 0.05 was considered statistically significant. Abbreviations: TNF, *tumor necrosis factor*; Control, (N = 6) *sham procedure*; CSD-Sham, (N = 7) *sham procedure with an α7nAChR-antagonist (Chlorisondamine)*; CSD-VGX, (N = 8) *cervical vagotomy with Chlorisondamine*.

A cervical vagotomy in combination with an α7nAChR-antagonist significantly increased lung injury compared to the corresponding control (sham) group (Figure 6). The median LIS was 0.416 (IQR 0.285–0.549) in the vagotomy with an α7nAChR-antagonist group versus 0.107 (IQR 0.055–0.233) (*p* = 0.004) in the control group. Between the control and sham with an α7nAChR-antagonist group, there were no statistically significant differences in the LIS (median 0.197 (IQR 0.091–0.361) (*p* = 0.242). The LIS was relatively higher in the vagotomy with an α7nAChR-antagonist compared to the sham with the antagonist group (*p* = 0.058). The macrophage cell count (×10^4^) in the BALF was higher in the vagotomy with the antagonist group compared with the control group (median 108 (IQR 81–125) versus 39 (IQR 31–79) (*p* = 0.045)), whereas the cell count of other inflammatory cells in the BALF and plasma did not differ (Figure 7). There were no statistically significantly differences in the inflammatory cell count in the BALF and plasma between the sham with an α7nAChR-antagonist and the controls. The TNF-α levels measured over time (preoperative, postoperative, 2 h postoperative and postmortem) in the serum did not differ between the groups (Figure 8). In this model, no reliable data on airway resistance and dynamic compliance were available due to premature deaths in the vagotomy with an α7nAChR-antagonist group (five of eight rats), most likely due to respiratory arrest.

Altogether, Model B further supported the finding that a cervical vagotomy aggravated lung injury and pulmonary functions. The administration of an α7nAChR-antagonist (Chlorisondamine diiodide) did not affect lung injury in the sham rats and it is unclear whether it further aggravated lung injury in the vagotomized rats, since a cervical vagotomy already aggravates lung injury as shown in model A.

### 3.3. Model C: Effect of High-Fat Enteral Nutrition on LPS-Induced Lung Injury

**Figure 9:**
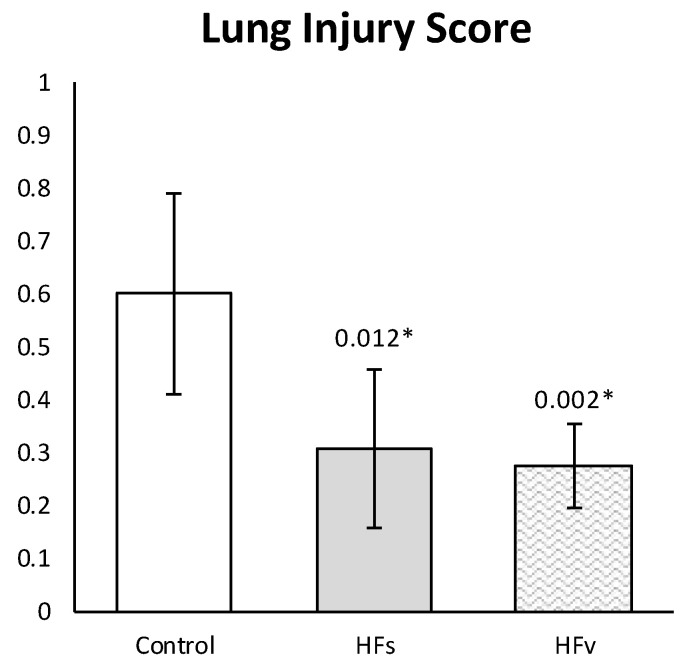
**Model C. Lung injury score.** Histopathological lung injury was lower in high-fat sham group, compared with the control group, even after a selective vagotomy. LIS between HFs and HFv groups did not differ (*p* > 0.999). Values are means with standard deviation. *p*-values as compared to the control group (after Bonferroni correction); a *p*-value < 0.05 was considered statistically significant (*). Abbreviations: Control, *fasting sham procedure*; HFs, *high-fat sham procedure*; HFv, *high-fat selective (abdominal) vagotomy*. N = 8 in each group.

Both in the sham and selective vagotomy group, high-fat nutrition attenuated the LIS, compared to the control (fasting sham) group (Figure 9). The mean LIS was 0.600 (±0.190) in the control vs. 0.307 (±0.150) in the high-fat sham (*p* = 0.012) and 0.275 (±0.079) in the high-fat (selective) vagotomy (*p* = 0.002) group, respectively.

**Figure 10:**
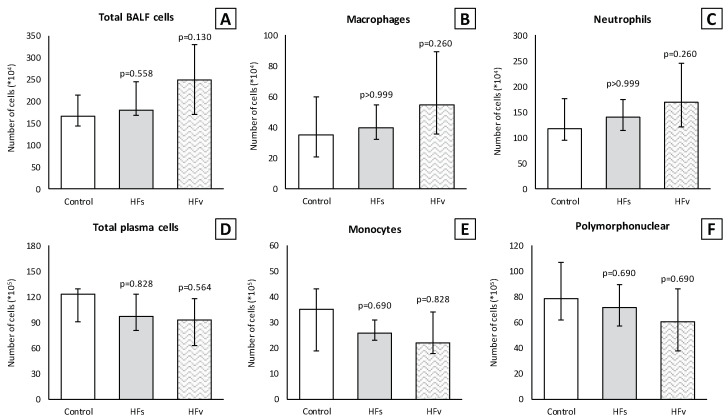
**Model C. Number of inflammatory cells in BALF (×10^4^) and plasma (×10^5^).** There were no statistically significant differences between groups in (**A**) total cell count, (**B**) macrophages and (**C**) neutrophils in BALF. Similarly, there were no differences between groups in (**D**) total cell count, (**E**) monocytes and (**F**) polymorphonuclear cells in plasma. Values are medians with interquartile range. *p*-values as compared to the control group (after Bonferroni correction); a *p*-value < 0.05 was considered statistically significant. Abbreviations: BALF, *broncho-alveolar lavage fluid*; Control, *fasting sham procedure*; HFs, *high-fat sham procedure*; HFv, *high-fat selective (abdominal) vagotomy*. N = 8 in each group.

**Figure 11:**
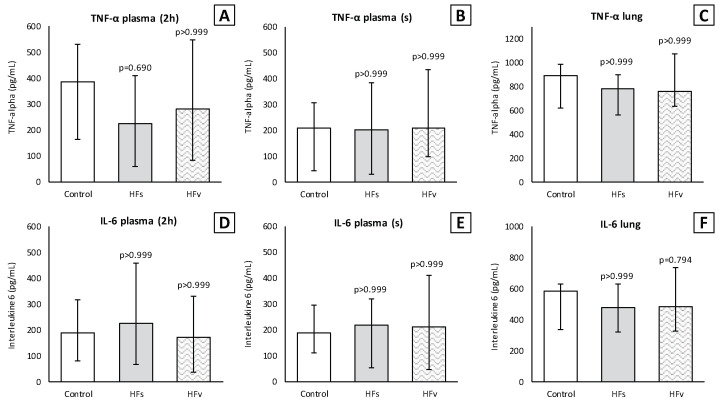
**Model C. Levels of TNF-α and IL-6 in plasma and lung homogenates (pg/mL).** Levels of TNF-α did not differ between the groups in plasma (**A**) at 2 h (2 h) after lipopolysaccharide (LPS) administration, (**B**) after rats were sacrificed (s) and (**C**) in lung homogenates. Similarly, levels of IL-6 did not differ in plasma (**D**,**E**) or lung homogenates (**F**). Values are medians with interquartile range. *p*-values as compared to the control group (after Bonferroni correction); a *p*-value < 0.05 was considered statistically significant. Abbreviations: TNF, *tumor necrosis factor*; IL, *interleukin;* Control, (N = 6) *fasting sham procedure*; HFs, (N = 6) *high-fat sham procedure*; HFv, (N = 4) *high-fat selective (abdominal) vagotomy*.

There were no differences in the inflammatory cell count (×10^4^) in the BALF between the control, high-fat sham and high-fat (selective) vagotomy groups, as shown in Figure 10. The total cell count was median 166 (IQR 144–215) vs. 181 (IQR 167–245) (*p* = 0.558); macrophages 35 (IQR 21–60) vs. 40 (IQR 32–55) (*p* > 0.999); and neutrophils 117 (IQR 96–177) vs. 140 (IQR 115–175) (*p* > 0.999), respectively. Similarly, the inflammatory cell count in the plasma (×10^5^) did not statistically significantly differ between the HFs and HFv group (Figure 10). Respectively, the total cell count was median 120 (IQR 75–134) vs. 97 (IQR 81–123) (*p* > 0.999); monocytes 35 (IQR 26–43) vs. 26 (IQR 23–31) (*p* = 0.254); and polymorphonuclear cells 76 (IQR 50–96) vs. 72 (IQR 57–90) (*p* > 0.999). The levels of TNF-α and IL-6 in the plasma at two hours after LPS administration, as well as in the plasma or BALF after sacrificing, were similar (Figure 10). Similarly, there were no significant differences between the TNF-α and IL-6 levels in lung homogenates (Figure 11).

Altogether, Model C showed that vagus nerve activation via high-fat nutrition attenuated histopathological lung injury, even after selective vagotomy.

## 4. Discussion

This experimental study demonstrated that a bilateral cervical vagotomy (proximal to the pulmonary branches) negatively impacted histopathological lung injury in a model of LPS-induced lung injury, whereas a selective vagotomy (below the pulmonary vagal branches) did not influence lung injury. Furthermore, a cervical vagotomy increased the macrophage cell count in the BALF compared to the controls and selectively sparing the vagus nerve, while the levels of the other inflammatory cells (e.g., neutrophils) in the BALF and serum and pro-inflammatory cytokines (i.e., TNF-α and IL-6) were not affected. This was in line with most previous studies, but the data are ambiguous [7,36,37,38]. Given the key role of the vagus nerve in inflammation, the finding that a cervical vagotomy did not affect cytokines or neutrophil influx was unexpected but could be attributable to the short time frame. Nevertheless, histopathological lung injury as measured by the LIS was more severe, which indicates that the inflammatory response to LPS was not limited to the CAIP.

Next to modulating inflammatory responses, the vagus nerve regulates several (protective) autonomic functions through its pulmonary fibers [7,8,11,12,13,14]. Specifically, pulmonary stretch receptors play an important role in the mediating pulmonary mechanics, such as influencing inspiratory and expiratory time in response to the changes in airflow and transpulmonary pressure [12,18,19,39]. This protective mechanism (Hering–Breuer reflex) prevents hyperinflation of the lungs, and it has been demonstrated that inhibiting it slows the respiratory rate while increasing the tidal volumes [12,18,19,39]. After a bilateral cervical vagotomy, this protective mechanism is markedly disrupted, as was observed by the change in the breathing pattern (i.e., slowing and deepening of respiratory movement) and increased dynamic compliance in the current study. This likely led to harmful tidal volumes and amplified lung injury [19,40]. Furthermore, disruption of the vagus nerve activity due to vagotomy induces neurogenic pulmonary edema [41]. Although this was not monitored in the current study, the vagotomized rats revealed central cyanosis (i.e., blue lips), which indicates that hypoxia in the lung tissue may have also contributed to lung injury. Importantly, a selective vagotomy did not affect lung injury or pulmonary functions, which indicates that the level of the vagotomy could be imperative. It may well be that transecting the vagus nerve below the pulmonary vagal branches during an esophagectomy can reduce the postoperative (pulmonary) complication rate [13,32].

Unexpectedly, the α7nAChR-agonist (GTS-21) did not influence LPS-induced lung injury in this specific experimental model, which was in contrast to previous studies [42,43,44]. However, in the current study, GTS-21 was only administered after a bilateral cervical vagotomy and not a sham procedure. Although GTS-21 increased pulmonary resistance—likely by directly activating the tracheal smooth muscle cells—the inhibitory effect on inflammation found in the non-vagotomized rats in previous studies might have been diminished due to the vagotomy. It could also be that lung injury was not solely mediated by the α7nAChR, but through other pathways. Specifically, administering an α7nAChR-antagonist (Chlorisondamine diiodide) did not influence lung injury in the sham rats, while its role in the vagotomized rats was unclear in the current study.

Additionally, high-fat enteral nutrition reduced histopathological lung injury even after a selective vagotomy. These current results are partly in line with previous studies involving hemorrhagic and endotoxic shock models, in which the administration of high-fat nutrition attenuated local and systemic inflammation via the vagus nerve [3,45,46,47,48,49,50,51]. In the current study, however, its beneficial effects occurred despite significant differences in the inflammatory cell count in the BALF and serum, as well as the cytokine levels (i.e., TNF-α and IL-6). As lipids enter the gastrointestinal tract, neuro-endocrine hormones such as Cholecystokinin (CCK) are released, which can bind to the CCK-receptors on vagal afferents, thereby releasing ACh [3,45,48,49,50,51]. The release of endogenous CCKs following the ingestion of lipids could have been insufficient in proportion to the dose of LPS administered exogenously in the current model. Previous studies either administered both LPS and selective CCK-agonists—thereby directly stimulating the CCK-receptors to inhibit the inflammatory response—or measured the endogenous levels in response to shock without administering LPS [3,45,46,47,48,49,50,51,52,53,54].

The CCK-receptors are also expressed in the rat lungs and are present on the pulmonary endothelial cells, macrophages, bronchial and alveolar epithelial cells [20,51,52,53,54]. Furthermore, there is evidence that CCKs can activate the autonomic nervous system via a humoral route [55,56]. Hence, it was unexpected that in the current study, both the local and systemic TNF-α and IL-6 levels were unaffected, and the inflammatory cell counts were similar between the feeding regimens, which could be attributable to the short time frame of the experiment. Nevertheless, histopathological lung injury was less severe after high-fat nutrition, which indicates that the modulatory effects of high-fat nutrition on LPS-induced inflammation in lung tissue may not be solely mediated by the autonomic nervous system or could already be present before the procedure.

These findings could support the implementation of a preoperative dietary intervention for patients undergoing an esophagectomy, as even after a selective vagotomy, lung injury was diminished, and such a preoperative intervention may potentially reduce (the severity of) postoperative pulmonary complications by activating anti-inflammatory pathways prior to surgery. To date, one randomized controlled trial investigating perioperative high-fat enteral nutrition has been performed in elective colorectal surgery but demonstrated no advantages on (pulmonary) complications [57]. However, pulmonary complications after colorectal surgery do not occur as frequently as in esophagectomy, and its pathophysiology may differ.

## 5. Conclusions

In conclusion, the current data show that the vagus nerve plays a pivotal role in modulating the inflammatory response and pulmonary function. High-fat nutrition reduced LPS-induced histopathological lung injury, but this was likely not solely mediated by the CCK-receptors on the vagus nerve or the vagus nerve efferents. As a dysregulated inflammatory response after an esophagectomy remains a critical issue, preserving and potentially stimulating the large pulmonary vagal branches could be a novel approach to reduce postoperative pulmonary morbidity after esophagectomy.

## Data Availability

The data presented in this study are available on request from the corresponding author. The data are not publicly available as approval from parties involved must be obtained for each case.
